# Current research on pharmacologic and regenerative therapies for osteoarthritis

**DOI:** 10.1038/boneres.2015.40

**Published:** 2016-03-01

**Authors:** Wei Zhang, Hongwei Ouyang, Crispin R Dass, Jiake Xu

**Affiliations:** 1 Center for Stem Cell and Tissue Engineering, School of Medicine, Zhejiang University, Hangzhou 310058, China; 2 School of Pathology and Laboratory Medicine, The University of Western Australia, Perth WA 6009, Australia; 3 School of Pharmacy, Building 306, Curtin University, Bentley, Perth WA 6102, Australia

## Abstract

Osteoarthritis (OA) is a degenerative joint disorder commonly encountered in clinical practice, and is the leading cause of disability in elderly people. Due to the poor self-healing capacity of articular cartilage and lack of specific diagnostic biomarkers, OA is a challenging disease with limited treatment options. Traditional pharmacologic therapies such as acetaminophen, non-steroidal anti-inflammatory drugs, and opioids are effective in relieving pain but are incapable of reversing cartilage damage and are frequently associated with adverse events. Current research focuses on the development of new OA drugs (such as sprifermin/recombinant human fibroblast growth factor-18, tanezumab/monoclonal antibody against β-nerve growth factor), which aims for more effectiveness and less incidence of adverse effects than the traditional ones. Furthermore, regenerative therapies (such as autologous chondrocyte implantation (ACI), new generation of matrix-induced ACI, cell-free scaffolds, induced pluripotent stem cells (iPS cells or iPSCs), and endogenous cell homing) are also emerging as promising alternatives as they have potential to enhance cartilage repair, and ultimately restore healthy tissue. However, despite currently available therapies and research advances, there remain unmet medical needs in the treatment of OA. This review highlights current research progress on pharmacologic and regenerative therapies for OA including key advances and potential limitations.

## Introduction

Osteoarthritis (OA), also known as degenerative joint disease, is characterized by cartilage degeneration and osseous overgrowth. OA is commonly encountered in today’s clinical practice. The incidence of OA increases with age, and it is one of the most prevalent diseases in older people. In the USA alone, 10% of men and 13% of women aged 60 and older have been diagnosed with knee OA.^1^ The symptoms of OA include joint pain, swelling, tenderness, stiffness, and sometimes locking, which may lead to disability and severely affect the life quality of patients.^2^

Due to the lack of self-healing capacity of articular cartilage, OA is among the most challenging joint diseases and there is currently no cure for it. The focus of treatment for OA is to reduce pain and improve function of the affected joints.^3^ Normally, applied pharmacologic therapy shows efficacy in pain relief but is frequently associated with adverse events.^4^ In recent years, emerging regenerative therapy has gained much attention as it can efficiently promote tissue repair and regeneration.^5^

This review mainly focuses on the current pharmacologic and regenerative therapeutic approaches for OA treatment. It includes therapy that has been confirmed clinically effective and used for decades, as well as therapy that shows promise in preclinical research and can potentially be translated for future clinical application, offering a systematic overview of progress in OA treatment in relation to progress with pharmacologic and regenerative therapy.

## Cartilage and OA

Articular cartilage is a typical hyaline cartilage that covers the ends of bones making up the joints in the body. It maintains smooth and frictionless movement, and dissipates stress in the joint. Articular cartilage is an avascular and aneural tissue; it consists primarily of chondrocytes and extracellular matrix including collagen type 2 and proteoglycans,^6^ which transmit loads, stabilize the matrix, and maintain a healthy cartilage microenvironment. Due to its load-bearing function, cartilage is highly susceptible to damage during sports activities, and wear and tear over time. First proposed by Hunter in 1742,^7^ it has long been recognized that cartilage defects cannot heal spontaneously. Researchers believe that the poor self-healing capacity is probably due to the poor blood supply and low metabolic activity in cartilage. If injured cartilage is not treated, it gets worse and affects surrounding tissue, and ultimately degenerates into OA.^8,9^

Microscopically, OA cartilage is characterized by loss of collagen and proteoglycans,^10,11^ thus perturbing the extracellular matrix structure and impairing the biomechanical properties.^6^ Chondrocytes near the superficial layer form clusters, whereas in the deep and calcified layers, they undergo apoptotic death.^12–14^ Chondrocyte proliferation is somewhat activated, but cannot resist the predominant catabolic activity.^12^ During disease progression, OA chondrocytes produce matrix-degrading enzymes including matrix metalloproteinase 13 (MMP13), which degrades collagen and A disintegrin, and metalloproteinase with thrombospondin motifs-5 (Adamts-5), which targets aggrecan.^15,16^ The synthesis of degradative enzymes further exacerbates the breakdown of articular cartilage. The biomechanical and biochemical changes would together disrupt cartilage homeostasis and contribute to the pathogenesis of OA, which leads to joint space narrowing, painful cartilage destruction, and loss of function.

**Figure 1:**
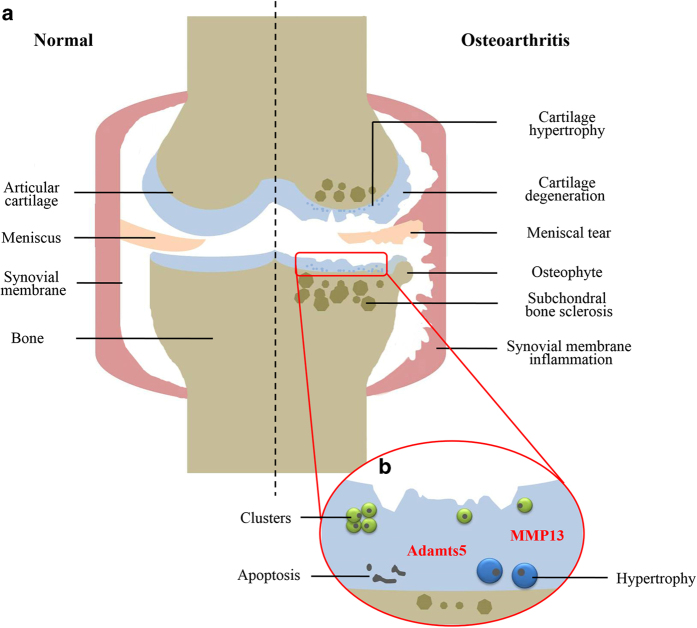
OA cartilage. (**a**) The changes of articular structure during OA progression. (**b**) Cellular responses in OA cartilage. OA, osteoarthritis.

In addition to cartilage degeneration, OA usually affects all structures in the synovial joint. Aberrant hypertrophy and calcification are reported in several OA cases, which is similar to the terminal differentiation process during endochondral ossification.^15,17,18^ Osseous outgrowths called osteophytes often form at the joint margins.^19,20^ Subchondral bone sclerosis,^21^ meniscal tear and extrusion,^22^ and synovial membrane inflammation (synovitis)^23^ may also occur due to the mechanical changes in OA cartilage, and make OA disease more debilitating (Figure 1).

## Overview of OA treatment

According to the Osteoarthritis Research Society International (OARSI) and the American Academy of Orthopaedic Surgeons (AAOS), the mainstay of OA treatments involves physical measures, drug therapy, and surgery.^3,24^ Physical therapy is a simple, everyday adjunctive treatment. Weight loss can adjust the imbalanced mechanical stress, lessen joint pain, and reduce OA risks.^25–27^ Moderate exercises help strengthen muscles and may delay the progression of OA.^28,29^ Alternative treatments such as spa, massage, and acupuncture are also beneficial but lack enough evidence to support efficacy.^30,31^ Surgery is only considered for severe cases when conservative therapy is ineffective because of the invasive trauma and higher risks. Arthroscopic irrigation and debridement provide a certain degree of pain relief but are not beneficial for long-term recovery.^32,33^ Drilling and microfracture techniques aim at penetrating the subchondral plate to induce bone marrow stromal cells for spontaneous repair, but the repaired tissue is inferior and consists of less durable fibrocartilage.^34,35^ Total joint replacement/arthroplasty is regarded as the best orthopaedic surgery for advanced OA. It can potentially reduce pain and improve joint function. Unfortunately, arthroplasty is not recommended for young patients, as the artificial implant has a finite lifespan (usually 10–15 years). In addition, the long-term results of arthroplasty differ significantly.^36,37^

Pharmaceutical therapy is the most commonly used OA treatment option aimed mainly at pain relief and anti-inflammation. The traditional OA drugs are limited to control OA symptoms, but none can reverse the damage in the OA joint. And, the traditional drugs are always overwhelmed by its high incidence of adverse effects. Studies of new OA drugs (mainly biologic agents) with more effectiveness and fewer side effects are underway. In addition, regenerative therapy holds the possibility of repairing and regenerating damaged or lost tissues to restore the original structure and function.^38^ It has already been applied in the orthopaedic clinic for several decades and promising outcomes have been achieved.^5,39^ In recent years, pilot clinical studies, although few, show the usefulness of regenerative therapy in the treatment of OA, suggesting its potential to be translated from bench to bedside.^40–47^ In the next sections, we would like to focus on the current pharmaceutical and regenerative therapies that have been clinically investigated, and that show safety and effectiveness in the management of OA.

## Pharmacologic therapy

### Traditional OA drugs

There are mainly five kinds of medications commonly used in today’s clinical treatment of OA: acetaminophen, non-steroidal anti-inflammatory drugs (NSAIDs), opioid analgesics, serotonin–norepinephrine reuptake inhibitors (SNRIs), and intra-articular injections. As there has been a considerable amount of literature focusing on the traditional drugs, here we provide a brief updated overview. The recommendations of the AAOS, American College of Rheumatology (ACR) and OARSI are summarized in Table 1.

#### Acetaminophen

Acetaminophen or paracetamol is an essential medicine as recognized by the World Health Organization, and is commonly used to reduce fever and relieve pains such as headache, muscle aches, backaches, and toothaches. Due to its relative safety and effectiveness, acetaminophen is recommended as the first-line oral analgesic for mild-to-moderate OA by most guidelines. According to ACR and OARSI guidelines, up to 4000 mg per day is an effective initial treatment for mild-to-moderate knee or hip OA.^3,48,49^ Overdosing acetaminophen may be toxic to the liver.^50,51^ Due to the risk of liver damage, on 13 January 2011, the US Food and Drug Administration (FDA) limited the amount of acetaminophen in prescription combination products to no >325 mg per dosage unit.^52^ Consistent with the change made by the FDA, the latest 2013 AAOS guideline downgraded the acetaminophen recommendation level to inconclusive and reduced the daily dosage from 4 000 to 3 000 mg.^24^ For patients with severe symptoms or who do not respond to acetaminophen, more potent drugs should be considered, such as NSAIDs.

#### NSAIDs

NSAIDs provide anti-inflammatory and analgesic effects, and have long been used as an important remedy for moderate-to-severe OA. Acetaminophen is not regarded as an NSAID as it has little anti-inflammatory effect. Some studies adopted meta-analysis to compare the safety and efficacy between acetaminophen and NSAIDs.^53,54^ By analyzing eight randomized controlled trials (RCTs), NSAIDs were better overall than acetaminophen in terms of pain relief. Although the efficacy of NSAIDs for OA treatment has been well documented, the health concerns, however, greatly restrict their extensive application. It is estimated that the occurrence of adverse effects is ~30% in people taking NSAIDs.^55^ A total of 1%–2% of people using NSAIDs develop gastrointestinal (GI) complications per year, which is much higher than that of people who do not use NSAIDs.^56,57^ Although selective COX-2 inhibitors appeared safer than traditional NSAIDs, several commercial drugs have been placed under scrutiny or withdrawn by the FDA. The first approved COX-2 inhibitor Celecoxib (Celebrex, Pfizer, New York, NY, USA) received an FDA alert for the potential risk of serious adverse cardiovascular events.^58^ Rofecoxib (Vioxx, Merck, Kenilworth, NJ, USA) and Valdecoxib (Bextra, Pfizer, New York, NY, USA) were withdrawn from the market for associated cardiovascular risks and other side effects.^59,60^ Therefore, there is a balance between the efficacy and safety of NSAIDs, and the benefit/risk ratio should be considered when taking these drugs. It is recommended by OARSI that NSAIDs be used at the minimum effective dose and prolonged use should be avoided as much as possible.^3^

#### Opioid analgesics

Opioids are used for the management of moderate-to-severe pain when NSAIDs and acetaminophen are ineffective or contraindicated.^3^ There has been an increased use of opioids in OA treatment (31% opioid prescribed in 2003 to 40% in 2009),^61^ however, the frequent adverse effects associated with opioids, including nausea, vomiting, dizziness, constipation, sleepiness, tiredness, and headache, may outweigh the benefits in pain relief.^62–64^ Opioid abuse is another potential risk of using these drugs. Routine use should be avoided, and low effective and tolerated doses are recommended.

#### SNRIs

SNRIs are primarily used in the treatment of depression and other mood disorders. In 2010, the FDA approved duloxetine, a selective SNRI, for the management of chronic musculoskeletal pain including OA.^65^ Duloxetine was considered an acceptable and favorable OA treatment based on the results from two double-blind, placebo-controlled RCTs.^66,67^ It may be a promising and efficacious way to alleviate OA pain for patients who are unable to take other commonly used drugs. The FDA approval and ACR recommendation^49^ also confirm its value in clinical use. However, AAOS and OARSI have not included duloxetine in their OA management guidelines,^3,24^ as more large-scale longitudinal studies to further investigate the safety and efficacy for OA treatment have to be performed.

#### Intra-articular injections

Intra-articular injection of corticosteroids and hyaluronic acid are selectively used in the treatment of OA. Corticosteroid injection is recommended by OARIS for patients with moderate-to-severe pain who do not respond to oral analgesic and anti-inflammatory agents.^3^ ACR and AAOS conditionally recommended corticosteroids for knee and/or for hip OA.^24,49^ Hyaluronic acid is a component in both healthy and OA joint fluid. Intra-articular injection of hyaluronic acid is recommended by OARIS as a treatment option for knee or hip OA.^3^ However, the efficacy of hyaluronic acid injection varies. The 2013 edition of the AAOS guideline downgraded the recommendation on hyaluronic acid from an inconclusive level to a non-affirming level after excluding the evidence of lower strength.^24^

### New OA drugs

The unsatisfactory effects and unacceptable side effects associated with traditional OA drugs warrant a continued search for potential new medications. Although few of them have received the regulatory approval for routine clinical use, a variety of new OA drugs have shown promising results in clinical trials (Table 2). On the basis of the potential therapeutic targets, they can be classified as chondrogenesis inducers, osteogenesis inhibitors, matrix degradation inhibitors, apoptosis inhibitors, and anti-inflammatory cytokines.^68^

#### Bone morphogenetic protein-7

Recombinant human bone morphogenetic protein-7 (BMP-7), also called osteogenic protein-1 (OP-1), was a FDA-approved biologic for the treatment of bone nonunions and spine fusion.^69^ A phase 1 safety and tolerability study first reported the use of BMP-7 in symptomatic knee OA.^70^ Thirty-three OA patients (mean age 60 years) were intra-articularly injected with four doses of BMP-7 or placebo. Participants who received 0.1 and 0.3 mg of BMP-7 showed greater symptomatic improvement and higher OARSI response rate. No dose-limiting toxicity was found. Phase 2 study with 0.1 and 0.3 mg dosing cohorts would be further conducted in future.

#### Interleukin-1β

Two randomized, double-blind, placebo-controlled studies attempted interleukin (IL)-1β inhibitor for knee OA treatment. One study administered IL-1β receptor antagonist intra-articularly in 160 patients,^71^ and the other injected AMG108, a IL-1β receptor antibody, subcutaneously and intravenously in 159 patients.^72^ Although IL-1β receptor antagonist/antibody was well tolerated, no significant clinical improvements were reported compared with placebo in either study.

#### β-Nerve growth factor

Tanezumab, a monoclonal antibody against β-nerve growth factor, has been tested clinically against OA. A proof-of-concept study of tanezumab was performed in 450 patients with knee OA.^73^ As compared with the placebo treatment, treatment with tanezumab significantly reduced knee pain while walking and improved the patients’ global assessment. However, 68% of patients receiving tanezumab were recorded with adverse events. Sixteen subjects developed rapidly progressive OA and required total joint replacements, prompting the FDA to request the suspension of the trials of tanezumab. However, from subsequent assessments, the risk of rapidly progressive OA with tanezumab was lower than that with tanezumab/NSAID combination therapy, and the rate of joint replacement was comparable between tanezumab monotherapy and placebo treatment.^74,75^ Therefore, the FDA has agreed to continue the clinical trials of tanezumab in OA treatment in conjunction with appropriate safety monitoring.

#### Fibroblast growth factor

The ideal biologic agents for OA treatment should alleviate pain, relieve symptoms and restore the normal structure of the joint. To date, no structure-modifying treatment has yet been approved. A proof-of-concept study has been conducted to evaluate the efficacy and safety of intraarticular sprifermin (recombinant human fibroblast growth factor-18) to treat symptomatic knee OA with 180 patients. Sprifermin treatment significantly reduced the loss of total and lateral femorotibial cartilage thickness and volume, as well as the joint space width narrowing in the lateral femorotibial compartment in a dose-dependent manner. No significant difference in serious adverse events was recorded between groups.^76^ More basic and clinical studies should be performed to fully investigate this novel OA biologic drug.

#### Platelet-rich plasma

Relatively more studies have been reported regarding platelet-rich plasma (PRP), which contains several kinds of growth factors including transforming growth factor β1 (TGF-β1), platelet-derived growth factor, vascular endothelial growth factor, insulin-like growth factor-1, and hepatocyte growth factor.^77^ Wang-Saegusa *et al.*^78^ treated 312 OA patients with total three intra-articular injections of autologous plasma rich in growth factors. After 6 months, statistically significant differences were seen in the following assessment instruments: visual analogue scale (VAS), SF-36, the Western Ontario and McMaster Universities Arthritis Index (WOMAC), and Lequesne Index. No adverse effects were observed. Positive trends and safety profile of PRP were also reported in other studies,^79,80^ suggesting a feasible and potential treatment for OA.

#### Human serum albumin

Ampion, a <5 kDa ultrafiltrate of human serum albumin, is currently being developed by Ampio Pharmaceuticals (Englewood, FL, USA) as an intra-articular injection to treat knee OA through suppressing pro-inflammatory cytokine production in T cells.^81,82^ In 2014, Ampio Pharmaceuticals completed the phase 3 clinical trial for Ampion and achieved ⩾40% improvement in WOMAC pain and function compared with placebo controls at 20 weeks.^83^ A subsequent multiple injection clinical study for severe knee OA was completed in 2015 and assured that multiple injections of Ampion were safe and effective,^84^ showing much potential for future treatment of OA.

#### Methotrexate

Methotrexate, a chemotherapeutic drug normally used to treat rheumatoid arthritis, is being tested in OA treatment. The first open-label pilot study with 30 patients treated with oral methotrexate was conducted in 2011 and indicated an analgesic efficacy for methotrexate in knee OA.^85^ Thirteen of 30 (43%) participants achieved ⩾30% reduction in VAS pain score and OARSI responder criteria. A pragmatic phase 3 RCT with anticipated 160 participants was also conducted by this group from 2014 to 2015.^86,87^ Although the study was completed, the actual efficacy has not yet been determined, as no published data were provided from this study.^87^ Another Egyptian group performed a randomized placebo-controlled trial with 144 patients to assess the efficacy of methotrexate in the treatment of symptomatic knee OA.^88^ After 28-week treatment, patients who received oral methotrexate showed significantly improvement in reducing pain and clinical synovitis compared with the placebo-treated group, indicating the dual benefit of methotrexate as a novel OA therapeutic option.

## Regenerative therapy

### Cell therapy

From the 1980s, cell-based therapy has been applied for cartilage repair and has rapidly developed over the past 30 years.^89^ It offers a long-term solution to repair and regenerate cartilage, alleviate symptoms and finally delay OA progression. Currently, cell therapy is applicable to both mature cells and stem cells.

#### Chondrocytes

First described by Brittberg *et al.*,^90^ autologous chondrocyte implantation/transplantation (ACI/ACT) is widely used in clinical practice and more than 15 000 patients have received this treatment worldwide.^91^ ACI mainly includes three key steps.^89,92^ First, a small mass of cartilage tissue (~150–300 mg) is collected from a healthy and less weight-bearing area during an arthroscopic biopsy procedure. Then, the extracellular matrix is enzymatically removed, and chondrocytes are isolated and cultured *in vitro* to acquire enough cells to reimplant. Finally, chondrocytes are implanted into the damaged area of the articular cartilage in a second open-knee procedure. On the basis of the various implantation methods, three generations of ACI have been developed in the past 20 years.^92^ The first generation adopts a piece of periosteum sutured over the prepared defect which is taken from the patient’s tibia. Then, the chondrocyte suspension is injected under the periosteum, where it forms a bioreactive chamber to allow cell growth and maturation.^90^ The limitations of the first generation lie in the periosteal delamination and hypertrophy, which lead to the development of the second generation of ACI using a bilayer collagen membrane. This biomaterial-based membrane is also sutured over the defect and followed by cell suspension injected underneath. The advance in tissue engineering contributes tangibly to the third generation. Cultured chondrocytes are pre-seeded on a three-dimensional scaffold and trimmed to fit the defect size. The ‘all-in-one’ composite is then implanted to the defect area with the fixation of fibrin glue. No periosteum or sutures are used in this method. Therefore, the third generation of ACI is also called matrix-induced autologous chondrocyte implantation/transplantation (MACI/MACT). MACI shows evident benefits over classic ACI as it reduces the surgical time, minimizes the fixation invasion and ensures even and long-term cell maintenance. More details about the scaffolds used in MACI will be discussed in the section ‘tissue engineering’.

The clinical outcomes of ACI have been well documented in full-thickness and osteochondral defect repair. In 1994, Brittberg *et al.* ^90^ first performed classic ACI in 23 patients with full-thickness cartilage defects. Eighty-eight percent of patients with femoral condylar defects showed good or excellent results after 2-year transplantation, but only 28.6% of patients with patella injuries had a satisfying outcome at the 3-year time point. Postoperative arthroscopy revealed 11 out of the 15 biopsies showing hyaline-like cartilage. Long-term follow-ups were subsequently reported by the same group for up to 10 years.^93,94^ Treatment for isolated femoral condyle defects and osteochondritis dissecans achieved ~90% good to excellent results. In addition, groups of patients with multiple and patella lesions demonstrated >65% good outcomes. Adverse effects were reported in 52 out of 101 patients.^94^ The most common complications were periosteal hypertrophy (26 patients) and intraarticular adhesions (10 patients). Overall, ACI can be regarded as a reasonable treatment for deep cartilage defects.

The commercial product Carticel (autologous cultured chondrocyte, manufacturer: Genzyme Biosurgery, Cambridge, MA, USA) was initially approved by the FDA in 1997 for the repair of symptomatic cartilage defects of the femoral condyle caused by acute or repetitive trauma.^95^ However, cartilage damage with generalized OA was an exclusion criterion for treatment.^96^ This is because ACI is applicable to localized cartilage defects surrounded by healthy cartilage. OA cartilage, however, often affects the adjacent areas and disturbs the homeostasis of the whole joint cavity. In this degenerative microenvironment, the implanted chondrocytes may undergo undesired dedifferentiation or apoptosis,^97,98^ therefore undermining efficacy. In recent years, two trials tested the first-generation ACI for the more challenging OA disease.^99,100^ Minas *et al.* ^100^ performed classic ACI on 153 patients with early OA changes with a mean age of 37.3 and average defect size of 4.9 cm^2^. At an average 5-year follow-up, treatment failure occurred in 12 knees that changed to joint arthroplasty. Among the patients considered without treatment failures, 92% experienced functional improvements, especially in the WOMAC pain and function scores. They concluded that ACI provided a plausible treatment for young OA patients and delayed the need for knee arthroplasty. Rosenberger *et al.* reported a case series of 56 patients older than 45 years, among which 32 patients were diagnosed with early degenerative changes (mean defect size 11.7 cm^2^).^99^ At the latest available follow-up, 72% of patients of all defect categories and 81% of OA patients experienced good or excellent clinical improvements. Three out of 32 OA patients were considered treatment failures. Their favorable findings pointed to consider classic ACI as a therapy for older age groups.

#### Mesenchymal stem cells

Although no severe clinical safety issues have been associated with the ACI technique, there are still some problems including the limited cells available, multiple surgical procedures involved, *in vitro* chondrocyte dedifferentiation, and donor-site morbidity caused by cartilage harvest.^101,102^ Mesenchymal stem cells (MSCs) are considered a potential cell source since they can be easily collected from various tissues such as bone marrow, adipose tissue, synovial membrane, and others, and have a high proliferation rate, chondro-differentiation capacity, and immunosuppressive activities.^103–105^

Bone marrow-derived MSCs (BM-MSCs) are the most attractive stem cells in regenerative medicine studies, and attempts have been made to use them for OA treatment. In 2011, Davatchi *et al.* ^40^ published a preliminary report of four patients with moderate-to-severe knee OA. Autologous BM-MSCs were cultured for 4–5 weeks, and 8×10^6^–9×10^6^ cells were injected into the knee joint. After 1-year follow-up, pain produced during walking was reduced in three patients. The number of stairs to climb to produce pain and pain on a VAS were improved in all four patients. As the physical parameters improved slightly, the results were encouraging, but not ideal. In another trail, Orozco *et al.* ^41^ performed MSC therapy on 12 patients diagnosed with Kellgren and Lawrence grades II to IV knee OA. More BM-MSCs (40×10^6^) were intra-articularly injected. One-year follow-up indicated marked increase in VAS (69%), Lequesne (65%), and WOMAC (78%) pain indices. Cartilage quality was significantly improved in 11 of 12 patients as evidenced by T2 mapping quantification.

Choi’s group tried to use adipose tissue-derived MSCs (AD-MSCs) to treat OA.^42,106^ They proposed that AD-MSCs had an advantage over BM-MSCs as obtaining cells from bone marrow is difficult and painful, together with risks of complications. They collected the cells from the infrapatellar fat pad and prepared these with PRP. Twenty-five patients with knee OA received this intra-articular injection. It yielded improved clinical outcomes on the 1-year follow-up as shown by the Lysholm, Tegner activity scale, and VAS scores; but no significant difference was detected between the MSC-treated group and the control group, which consisted of injections of PRP alone.^106^ On 2-year follow-up to their previous study,^42^ WOMAC, Lysholm, and VAS pain scores were as well significantly improved when compared with the preoperative status. Magnetic resonance imaging (MRI) examination further confirmed the improvement in cartilage. However, no comparison between the treatment and control groups was shown. Thus, one cannot distinguish the effect of AD-MSCs from that of PRP and accurately investigate the efficacy of AD-MSCs on OA treatment.

Overall, the preliminary results demonstrated that MSC-based therapy is encouraging in reducing pain and improving the function of OA. More RCTs with a large number of patients and long-term follow-up are needed before full-scale clinical translation.

### Tissue engineering

Tissue engineering involves the use of cells, scaffolds, and bioactive factors to enhance tissue mechanical properties and promote cell migration, attachment, proliferation, and differentiation to the desired cell type. Tissue engineering therapy has shown a lot of promising outcomes in the treatment of cartilage defects.^107,108^ For OA treatment, only a few results have been reported, though there is hope for the future.

#### Cell-based scaffolds

Treatment with cell-based scaffolds involves tissue harvest and cell expansion procedures that are used in ACI or other forms of cell therapy. The cells are pre-seeded on the scaffold, and the composite is subsequently implanted into the defect area with or without fixation. The third-generation ACI (MACI) is one of the most extensively used techniques for the clinical treatment of cartilage defects. Many commercial products have been approved for scaffold-associated chondrocyte implantation for more than a decade in Europe and Australia, such as Chondro-Gide (a bilayer collagen type 1/3 scaffold, manufactured by Geistlich Biomaterials, Wolhusen, Switzerland), Hyaff-11 (a hyaluronan-based scaffold, manufactured by Fida Advanced Biopolymers, Abano Terme, Italy), and BioSeed-C (a synthetic polymer scaffold composed of fibrin, polyglycolic/polylactic acid, and polydioxanone, manufactured by BioTissue, Zürich, Switzerland). In a case series published by Bauer *et al.*,^43^ 18 young patients suffering from medial knee OA (mean age 47 years) underwent high tibial osteotomy (HTO) combined with MACI using a collagen membrane scaffold (ACI-Maix Matricel GmbH, Herzogenrath, Germany). At the 5-year follow-up, the Knee Injury and Osteoarthritis Outcome Score (KOOS) was significantly improved. MRI results were improved at 24 and 48 months, but declined at the end point with only 33% good quality infill. No major complications but minor complications were found including patellar tendinitis. In another clinical trial, 79 patients with posttraumatic and focal OA cartilage defects were treated with autologous chondrocyte-seeded BioSeed-C scaffold. Clinical assessment was performed in 40 patients with 2-year follow-ups.^44^ The evaluated scores including International Knee Documentation Committee (IKDC) score, the Lysholm score, the Cincinnati knee score, and KOOS were all statistically significantly improved compared with preoperative values. Histological results showed good integration of the graft and newly formed cartilaginous tissue. In their subsequent 4-year follow-up with 19 patients of the cohort,^45^ the Lysholm score, IKDC score, and KOOS were further improved. MRI analysis revealed that 16 out of 19 patients experienced moderate to complete filling of the defects. These results showed that BioSeed-C is a potential therapeutic option for degenerative defects with stable effect. Although MACI technique has been reported with promising results for OA treatment in many trails, researchers demonstrated that MACI with Hyaff-11 scaffold was questionable for knee OA due to the poor performance and high failure rate. They treated 44 patients using MACI as a salvage procedure. After a 9-year mean follow-up, 27.3% treatment was considered to have failed. Almost half (47.7%) of the patients considered their condition unimproved and 39% would not choose this treatment again despite the significant improvement of IKDC and EQ-VAS scores. This long-term follow-up study indicated that the tissue-engineered cartilage implantation should be fully investigated before its application as a salvage procedure for the treatment of OA.

#### Cell-free scaffolds

Cell-free scaffolds are developed for one-stage procedure techniques, which can be either implanted alone to attract the endogenous cells or combined with biological products such as concentrated bone marrow or PRP.^98^ As exogenous cell transplantation is not required, it avoids the issues around the *in vitro* cell culture process, such as slow growth and aberrant differentiation.^102^ Clinical results of cell-free scaffolds on OA treatment are few. A case report documented a 46-year-old athletic patient with International Cartilage Repair Society (ICRS) grade IV degenerative chondral lesions treated with a three-layer nanostructured biomimetic scaffold (manufactured by Fin-Ceramica, Faenza, Italy) together with HTO.^46^ At 1-year follow-up, the patient was pain-free and returned to a satisfactory functional level. MRI analysis showed hyaline-like articular cartilage and non-visible subchondral oedema. An Italian group reported clinical improvements using PRP-enriched polyglycolic acid (PGA)-hyaluronan scaffold (chondrotissue, manufactured by BioTissue AG, Zürich, Switzerland).^47^ Fifty-two patients suffering degenerative chondral defects were treated, among which, 47 patients had grade I–III OA. The KOOS score was significantly increased, and histological staining revealed hyaline-like cartilage repair tissue at 1-year follow-up. The above two pilot studies confirmed the usefulness of cell-free scaffolds. However, an *in vivo* study using sheep OA model demonstrated that cell-free approaches were inferior to MACI by macroscopic and histological examinations.^109^ It remains to be seen whether cell-free scaffolds have more advantages over cell-seeded scaffolds in human studies.

#### Gene therapy

**Figure 2:**
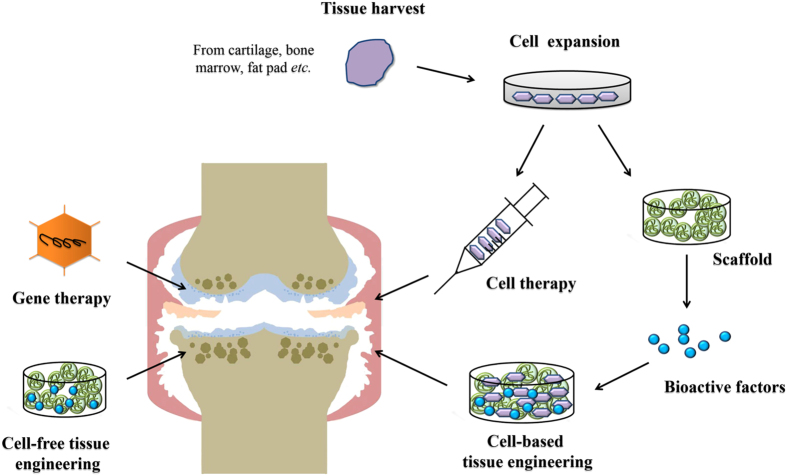
Current regenerative therapy for OA treatment. OA, osteoarthritis.

Gene therapy enables the spatiotemporal control and persistent synthesis of gene products at target sites. Several preclinical studies have confirmed its safety and efficacy, and implicated its prospects, but few clinical trials have been conducted and no gene products have been approved for OA treatment. At present, only TGF-β gene therapy has been clinically investigated in USA and Korea.^110^ This technique called TissueGene-C uses the retrovirally transduced allogeneic human chondrocytes overexpressing TGF-β1. Phase 1 and 2 trials have commenced, though results have not been published yet.^111,112^ From the published results of phase 1 study with 12 advanced OA patients,^113^ only some minor injection site reactions but no serious adverse events were observed after 1 year post dosing. Knee evaluation scores showed a dose-dependent improvement of symptoms. Phase 2 data, only available in abstract form,^114^ suggested a significant improvement in IKDC, WOMAC, and VAS scores without severe adverse events after 6 months. The placebo-controlled, double-blind, randomized phase 3 study was just completed on August 2015 but no study results have been posted as yet.^115^ The above-mentioned regenerative therapies are briefly summarized in Figure 2.

## Conclusion and future perspectives

In this review, we presented the current progress of pharmacologic and regenerative therapy for OA treatment. The traditional OA drugs are effective in reducing pain and inflammation but insufficient to slow, stop, or reverse the joint damage, and are frequently associated with adverse effects. New OA drugs such as biologic agents and chemotherapeutic drugs show more marked effects and fewer side effects, and look more promising than traditional OA drugs. Regenerative therapy is a novel strategy that has the potential to restore normal structure and function of damaged cartilage. At present, clinical studies in regenerative therapy are in its infancy with relatively rare and low-level evidence of success. Larger, random, controlled, and long-term follow-up studies are expected to take place in the coming years to confirm its safety and effectiveness. Although current pharmacologic and regenerative therapy show great promises, limitations still exist. Potential therapies may be developed by exploring more therapeutic targets and methods. The emerging targets that have been confirmed in preclinical animal studies are also summarized in Table 2.

### Inhibition of matrix degradation

As mentioned above, MMP13 and Adamts-5 are the main matrix-degrading enzymes that play a key role in the development of OA. In recent studies, MMP13 and Adamts-5 have been identified as downstream target genes involved in both β-catenin and TGF-β signaling pathways during OA development.^116,117^ Wang *et al.* ^118^ intraperitoneally injected CL82198, the MMP13 inhibitor in a murine model of injury-induced knee OA, which effectively decelerated OA progression, increased extracellular matrix production, and inhibited chondrocyte apoptosis. In another study performed by Chen *et al.*,^119^ Adamts-5 inhibitor (114810) and hyaluronic acid hydrogel were combined to treat rat OA knee joints and significantly prevented the progression of cartilage degeneration. In addition, Syndecan-4 was identified to control the activation of Adamts-5; therefore, the application of Syndecan-4-specific antibody could prevent proteoglycan loss and cartilage breakdown in a mouse OA model.^120^ However, the only clinical study with MMP inhibitor (PG-116800) for OA treatment resulted in termination due to musculoskeletal toxicity without clear benefit, suggesting more preclinical studies are needed to fully assess the safety and effectiveness of those matrix degradation inhibitors, and devise ways to improve efficacy.^121^

### Inhibition of hypertrophy and ossification

Current OA treatments aim to regenerate hyaline-like cartilage tissue. However, the repair tissue is often accompanied with undesirable chondrocyte hypertrophy and terminal differentiation, which cause matrix degradation and then impair the function of the repair tissue.^122,123^ It has been well identified that parathyroid hormone-related protein (PTHrP) acts in conjunction with Indian hedgehog to inhibit chondrocyte hypertrophy and regulates endochondral ossification through a negative-feedback loop.^124,125^ A recent study showed that systematic administration of recombinant human PTH (1–34; teriparatide, Forteo), the homolog of PTHrP could effectively inhibit cartilage degeneration and aberrant chondrocyte maturation in a surgically induced mouse OA model.^126^ In our own study, we found that intra-articular injection of recombinant human PTHrP (1–40) at 4–6 weeks post injury together with the implantation of collagen-silk scaffold significantly suppress chondrocyte terminal differentiation and promote chondrogenesis, therefore improving cartilage repair and regeneration in a rabbit osteochondral defect model.^123^ It is also reported that the inhibition of the hedgehog signaling could block the formation of hypertrophic chondrocytes and ameliorate OA development using small molecular inhibitors.^127,128^

### Target at subchondral bone

The therapeutic targets of most of today’s OA research are the articular cartilage itself, it is worthwhile to include the search of novel targets in the subchondral bone, which markedly becomes thicker and disrupted the mechanical stability in OA joints. Inhibition of TGF-β activity in subchondral bone may hold promise for OA treatment. Cao and colleagues reported in 2013 that injection of TGF-β type I receptor inhibitor (SB-505124) or the implantation of an antibody to TGF-β (1D11) in alginate beads could attenuate disease in ACLT-induced OA mice/rat.^129^ The Wnt/β-catenin signaling pathway has been demonstrated to be involved in both cartilage and bone development.^130^ Overexpression of dickkopf-related protein-1, one of the Wnt antagonists, ameliorated the severity of OA in mice by inactivation of the Wnt/β-catenin signaling in subchondral bone.^131^ These results not only suggested a potential treatment approach for OA disease but also shifted the treatment target from cartilage surface to subchondral bone, considering that OA is a disease of the whole joint.

### Pluripotent stem cells

Regarding cell therapy, pluripotent stem cells have unlimited self-renewal and chondrogenic differentiation capacity,^132^ offering an ideal cell source for cartilage repair and OA treatment compared with adult chondrocytes or MSCs. Embryonic stem cells (ESCs) are pluripotent stem cells derived from early mammalian embryos.^133^ ESC chondrogenesis can be achieved by *in vitro* culture supplemented with growth factors.^134,135^ ESCs have been reported to improve cartilage repair in animal models.^136,137^ In 2009, the US FDA approved the world’s first clinical trial with human ESCs for the treatment of spinal cord injury,^138^ making it possible to translate ESCs for OA disease in the future.

Induced pluripotent stem cells (iPSCs) are another type of pluripotent stem cells generated directly from adult cells. iPSCs are more applicable than ESCs, as they can be derived from more donor tissues with less immunorejection, and have less ethical controversy.^139^ iPSCs have been successfully induced to differentiate into various cell types including chondrocytes.^140–143^ Notably, Wei *et al.* generated iPSCs from human OA chondrocytes and then induced them towards chondrogenic differentiation, suggesting the potential of OA chondrocytes for OA treatment.^143^

### Endogenous cell homing

In terms of tissue-engineering strategies, more studies are now focusing on endogenous cell homing approaches. It aims at modifying a suitable microenvironment to recruit and mobilize the host cells from either the blood or a tissue-specific niche for self-repair. It avoids the costs, complexity, and risks involved in *in vitro* cell expansion and reimplant procedure, and is therefore regarded as a cost-effective and technically simpler alternative to current cell transplantation. The key factors to a successful cell homing process are the favorable cell niche that can be enhanced by excellent bioscaffolds, signaling biomolecules, and release technology.^144^ We have previously used a collagen type 1 scaffold containing stromal cell-derived factor-1 to create an *in situ* matrix environment.^145^ This microenvironment is conducive to the migration and adhesion of endogenous MSCs, thereby promoting the self-repair of partial thickness cartilage defects in a rabbit model. Another interesting study developed plasmid gene-activated osteochondral scaffold that could produce TGF-β1 for chondrogenic layer and BMP-2 for osteogenic layer.^146^ Endogenous BM-MSCs can be recruited and spatially controlled to simultaneously differentiate into chondro- and osteo-lineages within the scaffold. As OA usually affects complex tissues in the knee joint, this model may be exploited for future clinical treatment of OA disease.

## Figures and Tables

**Table 1:** Current recommended OA drugs by AAOS, ACR, and OARSI^3,24,48,49^

*Drugs*	*Recommendations*
Acetaminophen	AAOS: Inconclusively recommended for symptomatic knee OA with 3 000 mg per day (moderately recommended in the 2008 edition with up to 4 000 mg per day) ACR: First-line drug up to 4 000 mg per day OARSI: An effective initial oral analgesic for mild-to-moderate OA pain up to 4 000 mg per day
Non-selective NSAIDs	AAOS: Strongly recommended for symptomatic knee OA ACR: Conditionally recommended for hand, knee, and hip OA OARSI: Recommended for patients with symptomatic hip or knee OA at the lowest effective dose
Selective COX-2 inhibitors	AAOS: Strongly recommended for symptomatic knee OA ACR: Conditionally recommended for hand, knee, and hip OA OARSI: Recommended for patients with symptomatic hip or knee OA at the lowest effective dose
Opioid analgesics (tramadol)	AAOS: Strongly recommended for symptomatic knee OA ACR: Conditionally recommend for hand, knee, and hip OA OARSI: Consider use for the treatment of refractory pain in patients with hip or knee OA
SNRIs (duloxetine)	AAOS: Not included ACR: Conditionally recommended for patients ⩾75 OARSI: Not included
Intra-articular corticosteroids	AAOS: Inconclusively recommended for symptomatic knee OA ACR: Conditionally recommended for hip and knee OA OARSI: For patients with moderate-to-severe pain who are not respond to oral analgesic and anti-inflammatory agents
Intra-articular hyaluronic acid	AAOS: No longer recommended (inconclusively recommended in the 2008 edition) ACR: No recommendation OARSI: May be useful in patients with knee or hip OA

ACR, American College of Rheumatology; AAOS, American Academy of Orthopaedic Surgeons; NSAIDs, non-steroidal anti-inflammatory drugs; OA, osteoarthritis; OARSI, Osteoarthritis Research Society International; SNRIs, serotonin–norepinephrine reuptake inhibitor.

**Table 2:** New OA drugs and emerging therapeutics investigated in clinical studies (*) or preclinical animal studies

*Mode of action*	*Targets*	*Potential therapeutics*
Chondrogenic differentiation	BMP-7* FGF* PRP (containing several kinds of growth factors)*	rhBMP-7 (OP-1)^70^ rhFGF-18 (sprifermin)^76^ Autologous PRP^78–80^
Inhibition of hypertrophy and ossification	PTH/PTHrP receptor Hedgehog signaling	rhPTH (1–34) (teriparatide, Forteo),^126^ rhPTHrP (1–40)^123^ Smo inhibitor (HhAntag, LDE223)^127,128^
Inhibition of matrix degradation	MMP13 Adamts-5 Syndecan-4	MMP13 inhibitor (CL82198)^118^ Adamts-5 inhibitor (114810)^119^ Syndecan-4-specific antibody^120^
Inhibition of inflammation	IL-1β* HSA* Methotrexate*	IL-1β receptor antagonist,^71^ IL-1β receptor antibody (AMG108)^72^ a <5-kDa ultrafiltrate of HSA (Ampion)^83,84^ Methotrexate^85–88^
Reduction in pain	β-NGF*	Monoclonal antibody against β-NGF (Tanezumab)^73–75^
Subchondral bone	TGF-β Wnt/b-catenin	TGF-β type I receptor inhibitor (SB-505124), TGF-β antibody (1D11)^129^ Wnt antagonist (Dkk-1)^131^

β-NGF, β-nerve growth factor; BMP-7, bone morphogenetic protein-7; OP-1, osteogenic protein-1; Dkk-1, dickkopf-related protein-1; FGF, fibroblast growth factor; HSA, human serum albumin; PTH, parathyroid hormone; PTHrP, parathyroid hormone-related protein; rhBMP-7, recombinant human BMP-7; rhFGF; recombinant human FGF; rhPTHrP; recombinant human PTHrP; MMP13, matrix metalloproteinase 13; OA, osteoarthritis; PRP, platelet-rich plasma; TGF-β, transforming growth factor-β.
